# Antioxidant compounds of *Petasites japonicus* and their preventive effects in chronic diseases: a review

**DOI:** 10.3164/jcbn.20-58

**Published:** 2020-06-11

**Authors:** Miki Hiemori-Kondo

**Affiliations:** 1 Department of Food Nutrition, Tokushima Bunri University, 180 Nishihama, Yamashiro, Tokushima 770-8514, Japan

**Keywords:** *Petasites japonicus*, antioxidant activity, anti-allergy, neuroprotection, metabolic improvement

## Abstract

*Petasites japonicus* (*P. japonicus*) is a plant of the Asteraceae family. Its roots and stems have been used for the treatment or the prophylaxis of migraine and tension headache as a traditional Chinese medicine in Japan and Korea. Sesquiterpenoids, lignans, and flavonoids are components of *P. japonicus*. Regarding the biological activity of *P. japonicus*, its anti-allergic effect has been researched extensively using IgE antigen-stimulated degranulation of RBL-2H3 cells or passive cutaneous anaphylaxis reaction in experimental animal models. The study of the antioxidant activity of *P. japonicus* was initiated approximately 15 years ago using *in vitro* assays. In addition, its *in vivo* effect has also been examined in animal models with induced oxidative injury. Moreover, recently, many types of antioxidant compounds have been rapidly and simultaneously identified using the liquid chromatography–mass spectrometry technique. The number of reports on the other functions of this plant, such as its neuroprotective and anti-inflammatory effects, has been increasing. In this review, I summarized the studies of functional foods derived from *P. japonicus*, which may provide a basis for the development of potential functional foods. Finally, I discuss the future research avenues in this field.

## Introduction

*Petasites japonicus* (*P. japonicus*) is a plant of the Asteraceae family that is native to Japan. Sesquiterpens such as petasin and bakkenolides, fukinolic acid, lignans, and flavonoids (e.g., the aglycones of quercetin and kaempferol), are components of *P. japonicus*.^(1–6)^ The flower bud sprout of *P. japonicus* is a fukinoto and one of the wild plants that are harvested in spring. The flower buds and stems are used as foods in Japan and Korea. Moreover, the roots and stems of *P. japonicus* have long been used as a traditional Chinese medicine for the treatment and prophylaxis of migraine, tension headache, and spasms of the urogenital tract, gastrointestinal tract, and bile duct in East-Asian countries, such as China and Japan. In Europe and America, it is known as butterbur (*P. hybridus*), which has been reported to have effects on migraine,^(7–9)^ bronchial asthma,^(10)^ and seasonal allergic rhinitis and has been used as an herb.^(11–13)^ Therefore, the anti-allergic effect of *P. japonicus* has been researched extensively. Furthermore, the antioxidant activity of *P. japonicus* has been investigated and many active antioxidant compounds have been identified over the past 15 years. Moreover, its effects on chronic diseases have been demonstrated, suggesting its utility as a functional food. Studies have reported the physiological functions of *P. hybridus*;^(14,15)^ however, to the best of our knowledge, no review articles have particularly focused on the physiological functions of *P. japonicus*. Therefore, in this review, the functions of *P. japonicus* are summarized, as they may be useful for the development of potential functional foods.

## Varieties of Plants Grown in Japan

*P. japonicus* is a plant of the Asteraceae family and is native to Japan, and *P. japonicus* (Siebold et Zucc.) Maxim. is the only species of this family grown in Japan. It is harvested in all over Japan. *P. japonicus* is cultivated in Aichi, Gunma, Osaka, and a variety of “Aichi-wase-fuki” is widely distributed in Japan.^(16)^ Tokushima prefecture is a major production area for *P. japonicus* in South Japan, and three varieties, namely “Misato”, “Awaharuka”, and “Kamiyama-zairai”, are cultivated.^(17,18)^ Among them, Awaharuka has been cultivated for its high-quality flower buds, which has a suitable shape and tightly closed petals.

Furthermore, *P. japonicus* subsp. giganteus Kitam, a subspecies of *P. japonicus*,^(19)^ is cultivated in the northern area of the Kanto region; its leaves are very large and extend upward. Rawan-buki grows naturally in Hokkaido and is a kind of *P. japonicus* subsp. giganteus Kitam.^(20)^

## Antioxidant Compounds and *in vitro *Antioxidant Activity of *P. japonicus*

The antioxidant activity of the extracts from different tissues of *P. japonicus* was examined in various *in vitro* systems, such as the 1,1-diphenyl-2-picrylhydrazyl (DPPH) radical scavenging assay and ferric-reducing ability of plasma (FRAP) assays.^(21–23)^ Moreover, its antioxidant compounds were identified using a combination of an antioxidant assay with high-performance liquid chromatography (HPLC), liquid chromatography–tandem mass spectrometry (LC–MS/MS), and NMR techniques (Table 1). Matsuura *et al.*^(5)^ screened for antioxidative compounds in the flower buds of *P. japonicus subsp. gigantea Kitam* using the HPLC–DPPH method, and identified caffeic acid and several quercetin glucosides by HPLC coupled to a diode array detector, as well as ^1^H-NMR and flash desorption mass spectrometry analyses. In *P. formosanus*, petasiformin A was identified as a phenylpropenoyl sulfonic acid with DPPH radical scavenging activity.^(24)^ In *P. japonicus*, petaslignolide A is purified a new furofuran lignan with antioxidant activity.^(4)^ Kim *et al.*^(26)^ purified and isolated kaempferol as the active compounds of the stems of *P. japonicus*. The antioxidant activity of the active compound was examined by DPPH radical scavenging assay, thiobarbituric acid-reactive substance (TBARS) assay in the linoleic acid model system, and lipoxygenase inhibition assay.^(26)^ Moreover, several compounds such as caffeoylquinic acids and its isomer, quercetin, kaempferol glycosides, and fukinolic acid in the leaves and roots were identified. Among them, 3,5-di-*O*-caffeoylquinic acid exhibited the greatest radical-scavenging capacity, as assessed using an HPLC system with post-column online antioxidant detection based on 2-2'-azino-bis(3-ethylbenzothiazoline-6-sulfonic acid) (ABTS^+^) radical-scavenging activity.^(6)^ Lee *et al.*^(27)^ identified four flavonoids in *P. japonicus* leaves and reported that quercetin-3-*O*-*β*-d-glucoside, which was extracted among these flavonoids, showed the highest aldose reductase inhibitory activity on rat lens and was a potent agent against diabetic complications.

With the advancement of analyses and compound identification based on LC–MS/MS, antioxidant compounds have been identified rapidly using on-line HPLC–DPPH or on-line ABTS^+^. Choi *et al.*^(29)^ analyzed 10 components, including catechin, di-caffeoylquinic acid isomers, and naringenin, luteolin, liquiritin, kaempferol, and chrysoeriol derivatives and examined the antioxidant activity of extracts from the roots, stems, and leaves of Korean *P. japonicus* (Meowi) using DPPH, ABTS^+^, superoxide radical scavenging activities, and FRAP assays. Moreover, those authors also reported the anti-inflammatory effects of these compounds. We evaluated the antioxidant activity of an 80% ethanol extract of the flower buds of *P. japonicus using* oxygen radical absorbance capacity (ORAC) and DPPH radical scavenging activity. The ORAC values were attributed to H-ORAC; therefore, the trends in the results of the DPPH radical scavenging assay were consistent with those of the ORAC assay. Moreover, the antioxidative compounds that were determined using HPLC–DPPH methods and identified and quantified using LC–MS/MS included six antioxidant active compounds: caffeic acid, 3-*O*-caffeoylquinic acid [3-*O*-caffeoylquinic acid (chlorogenic acid)], fukinolic acid, and three di-caffeoylquinic acids (3,4-di-*O*-caffeoylquinic acid, 3,5-di-*O*-caffeoylquinic acid, and 4,5-di-*O*-caffeoyluinic acid). Fukinolic acid and 3,4-di-*O*-caffeoylquinic acid are major active compounds based on their activity and abundance.^(30)^ Conversely, Watanabe *et al.*^(25)^ reported that DPPH was epigallocatechin-3-*O*-gallate>fukinolic acid>chlorogenic acid and that the order of potency of the scavenging hydroxyl radical was epigallocatechin-3-*O*-gallate>fukinolic acid>gallic acid based on a mouse macrophage Raw 264.7 cell assay.

As mentioned above, the representative antioxidant components are caffeic acid, di-caffeoylquinic acid, fukinolic acid, and quercetin glycosides. The difference in their composition seems to depend on the tissue, the method of extraction, and the assay. Caffeic acid, caffeoylquinic acid, and quercetin glycosides are widely distributed in the plant kingdom, while fukinolic acid is specific to *P. japonicus*. The structures of fukinolic acid and fukiic acid in *P. japonicus* were reported by Sakamura *et al.*^(3)^ in 1973, which yield enzymatic browning substances by oxidation. Black cohosh (*Actaea racemose*) is used as an herb in America and Europe and is a member of the Ranunculaceae family that contains caffeic acid and fukinolic acid, which is a derivative of caffeic acid.^(31)^ *Cimicifuga heracleifolia* is also closely related to the genus *Actaea*. These plants contain fukinolic acid and cimicifugic acids,^(32,33)^ which are caffeic acid derivatives with documented antioxidant activities.^(33)^

Furthermore, the antioxidant activities of *P. japonicus* were examined using an *in vitro* assay with the cell lines Raw 264.7 and HCT-116, a human colorectal carcinoma cell line. Nitric oxide (NO) production was inhibited by fukinolic acid, as a main phenolic constituent in *P. japonicus*.^(25)^ Moreover, the polyphenolic extracts of leaves and roots exhibited anti-inflammatory effects by inducing the levels of the lipopolysaccharide-activated cyclooxygenase-2 (COX-2) and inducible nitric oxide synthase (iNOS) proteins.^(29)^ Conversely, its higher cytotoxic activity (IC_50_<25.0 µg/ml) against HCT-116 cells compared with that of *Angelica gigas* (34.75 µg/ml), *Erythronium japonicum* (44.06 µg/ml), and *Aster scaber* (54.87 µg/ml) has been shown.^(21)^ Moreover, based on an assay that used LLC-PK1 cells, an epithelial cell line of renal origin, it was shown that the ethyl acetate fraction of *P. japonicus* exhibited a high antioxidant activity via the upregulation of heme oxygenase 1 and thioredoxin reductases through the activation of the nuclear factor erythroid 2-related factor 2 (Nrf2) signaling pathway.^(28)^

## *In vivo* Antioxidant Activity of *P. japonicus*

With regard to oxidative stress *in vivo*, several examinations are performed (Table 2). Antioxidative effects of petaslignolide A or the butanol extract from the leaves of *P. japonicus* challenged with kainic acid have been reported in mouse brain based on TBARS value.^(34,35)^ Furthermore, improvement in seizure in kainic acid-treated mice by petaslignolide A has also been reported.^(4)^ In addition, antioxidant activities of the methanol extract of *P. japonicus* Max. have been demonstrated in monosodium l-glutamate-challenged mice.^(38)^ We performed two types of *in vivo* assays to evaluate the antioxidant activity of the flower bud extracts of *P. japonicus*.^(30)^ An animal model of Fe-nitrilotriacetate induced acute oxidative injury and mice fed with normal or high-fat diets were used as models of chronic disorders. The administration of the extracts orally to ICR mice prior to iron injection significantly suppressed the production of plasma TBARS, thus indicating that the flower bud extracts exert antioxidant effects under acute oxidative stress conditions. Moreover, the administration of these extracts at a concentration of 1% to C57BL/6 mice fed with high-fat diets for 16 weeks significantly decreased TBARS and triglyceride concentrations in the plasma of the mice, with no toxic symptoms. The effect of a methanol extract of *P. japonicus* on hepatotoxicity in rats induced by alcohol or carbon tetrachloride was also examined.^(36,37)^ The extract revealed protective effect and anti-lipid peroxidative effects in liver by decrease in glutamic oxaloacetic transaminase, glutamic pyruvic transaminase, and alkaline phosphatase, which is increased in the case of cardiovascular and biliary tract diseases. Cholesterol increased on liver cirrhosis and blood urea nitrogen directed post in liver function also decreased.^(37)^

In contrast, Han *et al.*^(39)^ have reported an increase in hepatic TBARS values and glutathione reductase and glutathione *S*-transferase activities and hepatic cytochrome mRNA expression following diets with 5% acetone extract of *P. japonicus* leaf powder, as revealed by the presence of pyrrolizidine alkaloids. Therefore, considering that a high amount of antioxidants were required to suppress the acute reaction, the amount of the toxic compound present in the *P. japonicus* flower bud extracts should be considered.

## Anti-Allergic Effect of *P. japonicus*

The anti-allergic effect of *P. japonicus* is well known at the research (Table 3). Regarding the former, RBL-2H3 cells from rats with basophilic leukemia with high-affinity IgE receptors are often used. The degranulation of IgE-antigen-stimulated RBL-2H3 cells leads to the release of *β*-hexosaminidase, similar to that observed for histamine and leukotriene. Therefore, *β*-hexosaminidase or its cytokine are measured and the inhibitory effect is examined. Yoshikawa *et al.*^(41)^ reported the degranulation inhibitory effect by fukinoside A from *P. japonicus*. Shimoda *et al.*^(42)^ examined the inhibitory effects of an aqueous ethanol extract of the aerial parts of Japanese *P. japonicus* and screened for active compounds. Several compounds, such as fukinones, caffeic acid, and di-caffeoylquinic acids, were identified as inhibitors. *In vivo*, the inhibitory effect of *P. japonicus* extracts on allergic reactions was examined using a passive cutaneous anaphylaxis (PCA) reaction on experimental guinea pig, rats, or mice.^(40,42,43)^ An ovalbumin-induced asthma model was also used to examine the anti-allergic effect of this plant. Recently, eremophilane lactone, a novel family of sesquiterpene compound, were isolated from *P. japonicus*. The product chemically modified from the lactone, 6β-angeloyloxy-3β, 8-dihydroxyeremophil-7(11)-en-12, 8β-olide, also inhibited the degrannudation on RBL2H-3 cells.^(46)^ The anti-allergic effects of bukkenolide B and petatewalide B from *P. japonicus* leaves were examined using an animal model.^(44,45)^ It is reported that they strongly inhibited the accumulation of eosinophils, macrophages, and lymphocytes in bronchoalveolar lavage fluid. In addition, petatewalide B increased the membrane potential of peritoneal macrophages C6 glioma cells. Therefore, it is suggested that petatewalide B has anti-allergic and anti-inflammatory effects.^(45)^ Moreover, petasitesin A and cimicifugic acid D inhibit the production of both prostaglandin E2 and NO, and petasitesin A inhibits the expression of iNOS and COX-2.^(47)^ Interestingly, it has been reported that petasitesin A and cimicifugic acid D exhibit strong affinities for both the iNOS and COX-2 enzymes, as assessed using docking studies. Thus, the basic studies on the anti-allergic effects of *P. japonicus* ingredients are mature; however, the effects have not been clinically confirmed. Conversely, it should be noted that allergic reactions to *P. japonicus* scapes have also been reported,^(48–52)^ and gastrointestinal sensitization with *P. japonicus* may have occurred due to non-heat-resistant allergens that cross-react with Asteraceae plant pollens, such as mugwort and ragweed pollens.^(48–53)^ However, because the allergen from *P. japonicus* is not known well, when using *P. japonicus* for anti-allergy and other functions, it would be necessary to respond individually.

## Neuroprotection by *P. japonicus*

Neuroprotective and anti-inflammatory activities are examined using *in vitro* assays with cell lines such as PC12 or B103 (Table 4). The neuroprotective effects of petaslignolide A isolated from *P. japonicus* leaves and of crude butanol extracts of *P. japonicus* leaves treated with kainic acid have been reported in the mouse brain.^(35,54)^ Moreover, the ethanol fraction and quercetin and kaempferol 3-*O*-(6''-acetyl)-*β*-glucopyranoside on β-secretase 1 (BACE1) production in B103 cells showed the presence of inhibitory activity and reducing the extracellular secretion of amyloid β (Aβ).^(55)^ Many patients with Alzheimer’s disease (AD) have deposition of Aβ in cortical blood vessels, leading to cerebral amyloid angiopathy. Aβ is directly responsible for the free radical production and lipid peroxidation, leading to apoptosis and cellular death. BACE1 is a key enzyme in the production of Aβ because of the deposition of the Aβ-peptide after proteolytic processing of the amyloid precursor protein by BACE1 and γ-secretase during the progression of AD. Therefore, BACE1 is a prime target for therapeutic intervention in AD. In addition, the suppression of reactive oxygen species (ROS) and the subsequent recovery of apoptotic cell death by the inhibition of Aβ-induced apoptotic cellular damage, ROS generation, and caspase-3 activity by kaempferol 3-*O*-(6''-acetyl)-*β*-glucopyranoside were reported.^(56)^ Kaempferol also showed neuroprotective effects on HT22 glutamate-induced oxidative stress cells by the regulation of the expression levels of Bcl-2, Bid, apoptosis-inducing factor, and mitogen-activated protein kinase (MAPK).^(58)^ The treatment with Japanese butterbur decreased Aβ levels *in vitro*.^(60)^ Moreover, the attenuate memory impairment and neuronal cell damage in Aβ-induced AD model using *P. japonicus* leaves was also demonstrated.^(61)^ The protective effects of sesquiterpenoids against neuronal cell death and its promoting effects on neurite outgrowth from PC12 cells have been reported.^(57,59)^ Recently, protein aggregation has been described as the principal component of numerous protein misfolding pathologies termed proteinopathies, such as AD, Parkinson’s disease, prion diseases, and AA amyloidosis with treatment needs. An automated real-time microliter-scale high-throughput screening system for amyloid aggregation inhibitors using quantum-dot nanoprobes that can simultaneously screen multiple samples was developed and *P. japonicus* was assessed.^(63)^ However, subsp. giganteus seemed to have low inhibitory effects. On the other hand, the anti-neuroinflammatory effects of petatewalide B on lipopolysaccharide-stimulated microglia and its mechanism underlying AMP-activated protein kinase (AMPK)/Nrf 2-signaling pathway have been reported.^(62)^

## Metabolic Improvement by *P. japonicus*

There are few reports of anti-obesitic and anti-adipogenic activities (Table 5). Han *et al.*^(64)^ reported that high-fat diet containing 3% chikusetsusaponins isolated from *P. japonicus* rhizomes significantly increased the fecal content and triacylglycerol level in rats at day 3. In addition, orally administered chikusetsusaponins also exhibited inhibition in the elevation of the plasma triacylglycerol and the pancreatic lipase activity, delaying the intestinal absorption of dietary fat. Lee *et al.*^(66)^ demonstrated the inhibitory activity of pancreatic lipase in leaf and stem *in vitro*. Watanabe *et al.*^(65)^ have reported that the administration of diets comprising *P. japonicus* ethanol extracts resulted in a decrease in weight gain, visceral fat accumulation, plasma cholesterol, and glucose concentrations in mice fed with a high-fat diets. Its energy expenditure is reported to be upregulated by flavonoids, such as quercetin.^(69)^ The mechanism consists in the suppression of preadipocyte differentiation/three adipogenetic transcription factors, the peroxisome proliferator-activated receptor (PPAR) γ, the CCAAT enhancer-binding protein, and the sterol regulatory element-binding protein 1C, with a decrease in body weight, gain and accumulation of visceral fat tissue, and amelioration of the plasma cholesterol concentration. Adachi *et al.*^(67)^ reported that petasin modulates glucose metabolism and activates AMPK through the inhibition of mitochondrial respiration. Moreover, *S*-petasin isolated from *P. japonicus* extracts yielded reduction of glucose uptake and inhibition of triglyceride accumulation by inhibiting the PPAR-γ signaling pathway in the 3T3-L1 cell line. These results indicate that *S*-petasin has anti-adipogenic activity.^(68)^ Based on this information, petasin is thought to be a representative candidate for the regulation of obesity. However, the mechanism underlying the improvement of metabolic syndrome and obesity is limited by the uptake of glucose and the activation of AMPK. Moreover, *S*-petasin is the only active compound identified as anti-obesitic in *P. japonicus*. Nevertheless, it has been reported that caffeic acid and chlorogenic acid increase body weight, lipid metabolism, and obesity-related hormone levels in mice fed with high-fat diets.^(70)^ Because many compounds occur in *P. japonicus*, as shown in Table 1, the identification of other mechanisms and active compounds are needed for the management of metabolic syndrome.

## Anti-Cancer Effect of *P. japonicus*

Reports on the anti-cancer effects of this plant are scarce (Table 6). Picrasinoside B isolated from *Picrasma quassioides* inhibited tumor growth and showed antitumor activity against P-388 lymphocyte leukemia cells.^(71)^ In addition, fukinolide isolated from *P. japonicus* showed antitumor activity; however, it was not as strong as that observed by picrasinoside B. Petasiphenol, a polyphenol from *P. japonicus*, inhibited DNA polymerase λ, suggesting it to be a potent antiangiogenic agent.^(72)^ The growth inhibition afforded by the methanol extract occurs via the inhibition of the Akt/mTOR and Wnt signaling pathways in Hep3B hepatocellular carcinoma (HCC) cells, suggesting that the extract has an antiproliferative effect.^(73)^ Hwang *et al.*^(74)^ reported the induction of apoptosis by *P. japonicus* ethanol extract in cervical carcinoma HeLa cells. Although there are some reports of the apoptotic effect of *P. japonicus* extracts, there is little information on their antitumor activity.

## Possible Adverse Effects of *P. japonicus* and Attention to Pyrrolizidine Alkaloids

As described above, Han *et al.*^(39)^ reported an increase in hepatic TBARS values after diets including a 5% acetone extract of *P. japonicus* leaf powder, as revealed by the presence of pyrrolizidine alkaloids. Pyrrolizidine alkaloids are toxic and can cause liver damage and cancer.^(75–77)^ Several types of pyrrolizidine alkaloids have been identified that are mainly found in plant families such as Asteraceae, Aabaceae, and Oraginaceae. Pyrrolizidine alkaloids in *P. japonicus* comprise mainly petasitenine, neopetasitenine, and senkirukin, while mass signals corresponding to them were not detected.^(30,42)^ Furthermore, the comparison of the liver and kidney weights of C57BL/6 mice administrated 1% *P. japonicus* flower bud extracts for 15 weeks with those of non-treated mice revealed an absence of differences; moreover, a disorder of appearance was not observed.^(30)^ However, the concentrations of pyrrolizidine alkaloid are not sufficient for causing acute poisoning in most cases. Therefore, the intake of such extracts may be considered safe for humans. However, because some adverse effects of the absorption of pyrrolizidine alkaloid have been reported, as described above, attention must be paid to the use of large amounts of the extract at once individually, particularly for patients with diseases, pregnant women, or children.^(78–80)^ Conversely, the concentrations of pyrrolizidine alkaloids can be decreased by boiling and simmering the plant in tap water.^(80)^ Therefore, the reduction of the concentrations of pyrrolizidine alkaloid is recommended before the consumption of the stems or flower buds of *P. japonicus*.

## Conclusion

In this review, I described the potential pharmacological efficacy of *P. japonicus* extracts or its isolated compounds, such as polyphenols and sesquiterpenes. It can also be a useful bioresource in the production of functional ingredients. However, the bioactive compounds of this plant have not been explored in detail *in vivo*, except for the antioxidant activity of petaslignolide A in the brain, usefulness of petatewalide B in anti-asthma, and activities of petasin and chikusetsusaponins in improvement of the metabolism of fat and glucose. *In vivo* examinations were primarily performed using plant powder or crude extracts. Therefore, it is important to identify and purify active compounds for its functional utilization. In particular, it would be interesting to elucidate the *in vivo* effects of bioactive compounds that exist only in *P. japonicus*.

Studies focusing on neuroprotective and anti-inflammatory functions have been increasing, indicating increased concern toward anti-aging to prolong healthy life expectancy. Some mechanisms underlying neuroprotection have been elucidated and the AD preventive effect of *P. japonicus* or its derived compounds is expected. However, few *in vivo* examinations on this function have been conducted; hence, further studies are required to elucidate their bioactivities. Moreover, *in vivo* studies for anti-obesity and anti-cancer effects are necessary for health promotion and prevent of disease. This requires comparison with other plants and active compounds to exhibit its predominance. In addition, clinical trials on some functions, including anti-allergic effects, have been conducted for *P. hybridus*, but few have been conducted for *P. japonicus*. We must also consider the concerning adverse effects of pyrrolizidine alkaloids. When using several active compounds in crude extracts, we must ensure that there is no contamination of pyrrolizidine alkaloids. In the future, we must conduct clinical trials for the utilization of these *P. japonicus* effects; if we can obtain beneficial effects without adverse events in the trial, it may be used as a safe food material or pharmacological source.

## Figures and Tables

**Table 1:** Analysis and identification of antioxidant compounds in *P. japonicus*

Assay	Compound (Source, part, and fraction)	Author	Ref.
HPLC–DPPH	Quercetin 3-*O*-*β*-d-glucoside, quercetin 3-*O*-*β*-d-6''-*O*-acetylglucoside, rutin, caffeic acid (70% ethanol extraction of *P. japonicus* subsp. *gigantea* Kitam. flower bud)	Matsuura *et al.* (2002)	(5)
DPPH radical scavenging assay	Petasiformin A (leaves of *P. formosanus* KITAMURA)	Lin *et al.* (2004)	(24)
DPPH radical scavenging assay	Petaslignolide A [*n*-butanol fraction of the methanolic extract of *P. japonicus* (Sieb. et Zucc.) Maxim. leaves]	Min *et al.* (2005)	(4)
Scavenging superoxide anion, NO, DPPH, radical scavenging, Raw 264.7	Chlorogenic acid, fukinolic acid, 3,5-dicaffeoyl quinic acid, and 3,4,5-tricaffeoyl quinic acid (leaves of *P. japonicus* Fr. Schmidt)	Watanabe *et al.* (2007)	(25)
DPPH radical scavenging assay, TBARS in the linoleic acid model system, lipoxygenase inhibition assay	Kaempferol (*P. japonicus* stem)	Kim *et al.* (2008)	(26)
HPLC system with post-column online antioxidant detection based on ABTS^+^ radical scavenging activitiy	5-Caffeoylquinic acid, fukinolic acid, 3,5-di-*O*-caffeoylquinic acid, quercetin-3-*O*-(6''-acetyl)-*β*-glucopyranoside, 4,5-di-*O*-caffeoylquinic acid, and kaempferol-3-*O*-(6''-acetyl)-*β*-glucopyranoside (methanol extract *of P. japonicus* leaves and roots)	Kim *et al.* (2012)	(6)
Aldose reductase inhibition on rat lenes	Kaempferol-3-*O*-(6''-acetyl)-*β*-d-glucoside, quercetin-3-*O*-(6''-acetyl)-*β*-d-glucoside, kaempferol-3-*O*-*β*-d-glucoside, quercetin-3-*O*-*β*-d-glucoside (methanol extract of *P. japonicus* leaves)	Lee *et al.* (2015)	(27)
Scavenging activity against superoxide anion radical, LLC-PK1 cells	Ethyl acetate extract of *P. japonicus* (high polyphenol and flavonoid content)	Kim *et al.* (2016)	(28)
DPPH scavenging activitiy, ABTS^+^ scavenging activitiy, superoxide radical scavenging activity, FRAP assays, RAW 264.7	3,5-Dihydroxy-7,3',4',5'-tetramethoxy flavanonol hydroxy feruloyl glucoside, dicaffeoylquinic acid, naringenic hexoside, luteolin-7-*O*-[6'-dihydrogalloyl]-glucosyl-8-*C*-pentosyl-glucoside, liquiritin, 3,4-di-*O*-caffeoylquinic acid, 1,3-*O*-dicaffeoylquinic acid hexoside, kaempferol-3-*O*-acetylglucoside, chrysoeriol-methyl ether (Korean *P. japonicus* leaves, stems, and roots)	Choi *et al.* (2017)	(29)
HPLC–DPPH, ORAC	3-*O*-Caffeoylquinic acid, fukinolic acid, 3,4-di-*O*-caffeoylquinic acid, 3,5-di-*O*-caffeoylquinic acid, and 4,5-di-*O*-caffeoylquinic acid (80% ethanol extract of *P. japonicus* (Sieb. et Zucc.) Maxim. flower bud)	Hiemori-Kondo *et al.* (2020)	(30)

HPLC–DPPH, high performance liquid chromatography-1,1-diphenyl-2-picrylhydrazyl; NO, nitric oxide; TBARS, thiobarbituric acid-reactive substance; ABTS^+^, 2-2'-azino-bis(3-ethylbenzothiazoline-6-sulfonic acid); FRAP, ferric-reducing ability of plasma; ORAC, oxygen radical absorbance capacity.

**Table 2:** *In vivo* antioxidant activity of *P. japonicus* and its derived compounds

Animal model	Effect and mechanism	Source, part (fraction), and compounds	Author	Ref.
Kainic acid-challenged mice	Restore TBARS values and cytosolic GSH levels in the brain	*P. japonicus* butanol extract (400 mg/kg) gavage for 4 days	Oh *et al.* (2005)	(34)
Kainic acid-challenged mice	Restore TBARS values and cytosolic GSH levels in the brain	Petaslignolide A in *P. japonicus* (Sieb. et Zucc.) Maxim. leaves (40 mg/kg for 4 days)	Cui *et al.* (2005)	(35)
Kainic acid-treated mice	Antioxidant and antiseizure activities	Petaslignolide A in *P. japonicus* (Sieb. et Zucc.) Maxim. leaves (50 mg/kg for 4 days)	Min *et al.* (2005)	(4)
Alcohol-treated Sprague-Dawley rats	Suppression in the decrease in AST activity, suppression or increase in the hepatic activities of catalase and GSH-Px, and increase in GSH levels	Ethanol extract of *P. japonicus* (Sieb. et Zucc.) Maxim. leaves and stems (200 mg/kg/day)	Cho *et al.* (2007)	(36)
CCl_4_-induced lipid peroxidation, hepatotoxicity in rats	Increase in anti-lipid peroxidative effects and decrease in the levels of GOT, GPT, ALP, BUN, and cholesterol	Methanol extract of *P. japonicus* (1.0 g/kg)	Park (2007)	(37)
Monosodium l-glutamate-treated ICR mice	Improvement in plasma lipid profiles and decrease in oxidative stress by the upregulation of hepatic antioxidant enzymes	The butanol fraction from the methanol extreact of butterbur (*P. japonicus* Max.) leaves (0.1% or 0.3% for 1 week and on day 7)	Park *et al.* (2010)	(38)
F344/DuCrj rats	Increased liver weight, increased TBARS and glutathione levels in the serum and liver, and hepatic GR and GST activities	*P. japonicus* leaves and its acetone extract (5% leaf powder for 4 weeks)	Han *et al.* (2012)	(39)
Iron-induced oxidative ICR mice, plasma TBARS of C57BL/6 mice fed with a high-fat diet	Suppression in plasma TBARS production in ICR mice, plasma TBARS, and decrease in triglyceride concentrations in C57BL/6 mice	*P. japonicus* (Sieb. et Zucc.) Maxim. flower bud (80% ethanol extract) (8 g of powder base/kg or 1% for 16 weeks)	Hiemori-Kondo *et al.* (2020)	(30)

TBARS, thiobarbituric acid-reactive substance; GSH, glutathione; AST, aspartate aminotransferase; GSH-Px, glutathione peroxidase; CCl_4_, carbon tetrachloride; GOT, glutamic oxaloacetic transaminase; GPT, glutamic pyruvic transaminase; ALP, alkaline phosphatase; BUN, blood urea nitrogen; GR, glutathione reductase; GST, glutathione *S*-transferase.

**Table 3:** Anti-allergic effect

Assay	Effect and mechanism	Source and compounds	Author	Ref.
Guinea pig PCA	Antihistaminic and anti-allergic activities	6β-Hydroxyeremophilenoide and 6β,8-dihydroxyeremophilenolide from the rhizomes of *P. japonicus* Maxim. var. *giganteus* Hort.	Tobinaga *et al.* (1983)	(40)
RBL-2H3 mast cells	Inhibition of *β*-hexosaminidase release; degranulation	Fukinoside A from *P. japonicus* Maxim.	Yoshikawa *et al.* (2006)	(41)
IgE-sensitized RBL-2H3 cells, rat PCA reaction, a guinea pig trachea strip	Inhibition of *β*-hexosaminidase release (leukotriene C4/D4/E4 synthesis and TNF-α production) and PCA reaction and suppression of smooth muscle constriction induced by histamine and leukotriene D4	70% Ethanol extract from aerial parts of Japanese butterbur, (+)-fukinone, caffeic acid, 2β-hydroxyfukinone, chlorogenic acid, fukinolic acid, 4,5-dicaffeoylquinic acid, 3,5-dicaffeoylquinic acid, 4,5-dicaffeoylquinic acid methyl ester, and dotorioside II	Shimoda *et al.* (2006)	(42)
IgE-sensitized RBL-2H3 cells, mouse PCA reaction	Inhibition of IgE antigen-stimulated degranulation, TNF-α and IL-4 cytokine expression and transcription factor NF-κB, IgE-antigen-induced PCA reactions	Ethyl acetate extract from fermented *P. japonicus* leaves	Bae *et al.* (2009)	(43)
RBL-2H3 mast cells, peritoneal macrophages, ovalbumin-induced asthma model	Inhibition of degranulation, gene inductions of iNO synthase and COX-2	Bakkenolide B from *P. japonicus* (Sieb. et Zucc.) Maxim. leaves	Lee *et al.* (2013)	(44)
RBL-2H3 mast cells, C6 glioma cells, ovalbumin-induced asthma model	Supression of *β*-hexosaminidase and fluorescence change of Ca^2+^, inhibition of iNOS induction, NO production, and accumulations of eosinophils, macrophages, and lymphocytes	Petatewalide B from *P. japonicus* (Sieb. et Zucc.) Maxim. leaves	Choi *et al.* (2016)	(45)
RBL-2H3 mast cells	Inhibition of degranulation activated via the high affinity IgE receptor, FcɛRI	6β-Angeloyloxy-3β,8-dihydroxyeremophil-7(11)-en-12,8β-olide	Qian *et al.* (2016)	(46)
RAW264.7 macrophages, docking studies	Inhibition of the production of both PGE2 and NO, expressions of iNOS and COX-2, and high affinity with iNOS and COX-2	Boild water extract of the leaves of *P. japonicus*, petasitesin A and cimicifugic acid D	Lee *et al.* (2019)	(47)

PCA, passive cutaneous anaphylaxis; iNOS, inducible nitric oxide synthase; COX-2, cyclooxygenase-2; NO, nitric oxide; PGE2, prostaglandin E2.

**Table 4:** Neuroprotection and anti-inflammatory activities

Assay	Effect and mechanism	Source and compounds	Author	Ref.
ICR mice challenged with kainic acid	Prevention of oxidative brain damage (attenuation of the neurobehavioral signs and neuronal loss in the hippocampal)	Butanol fraction and subfraction from the methanol extract of *P. japonicus* leaves	Sok *et al.* (2006)	(54)
APP695-transfected B103 cells	Inhibition of BACE1 activity and reduction in extracellular Aβ secretion	Ethyl acetate fraction of *P. japonicus*, quercetin and kaempferol 3-*O*-(6''-acetyl)-*β*-glucopyranoside	Song *et al.* (2008)	(55)
Mouse neuroblastoma B103 cells	Inhibition of Aβ-induced apoptotic cellular damage, ROS generation, and caspase-3 activity	Kaempferol 3-*O*-(6''-acetyl)-*β*-glucopyranoside from *P. japonicus* leaves	Song *et al.* (2012)	(56)
Human dopaminergic SH-SY5Y cells treated with cobalt chloride	Neuroprotective activity against neuronal cell death of five sesquiterpenes	Ten sesquiterpenes isolated from the whole *P. japonicus* plant	Wang *et al.* (2013)	(57)
Hippocampal neuronal HT22 cells with glutamate-induced oxidative stress	Regulation of the expression levels of Bcl-2, Bid, AIF, and MAPK	Kaempferol from the stems of *P. japonicus*	Yang *et al.* (2014)	(58)
PC12 cells derived from the adrenal gland of rattus norvegicus	Promotion of neurite outgrowth	Eighteen sesquiterpenoids isolated from edible *P. japonicus*	Xu *et al.* (2016)	(59)
Gel electrophoresis and ELISA	Decrease in the Aβ levels	Five plant sprouts’ extracts including Japanese butterbur	Okada *et al.* (2016)	(60)
HT22 cells and Aβ_25–35_ plaque-injected AD mouse models	Protection of neurons against Aβ_25–35_ plaque-injected neurotoxicity	Boiled water extract from *P. japonicus leaves*	Kim *et al.* (2018)	(61)
Lipopolysaccharide-stimulated microglia	Alleviation of IL-1β, IL-6, and TNF-α production and up-regulation of HO-1 and NQO1 via the AMPK/Nrf2-signaling pathway	Petatewalide B from *P. japonicus*	Park *et al.* (2018)	(62)

APP, amyloid precursor protein; BACE1, β-secretase 1; Aβ, amyloid β; ROS, reactive oxygen species; AIF, apoptosis-inducing factor; MAPK, mitogen-activated protein kinase; ELISA, enzyme-linked immunosorbent assay; AD, Alzheimer’s disease; HO-1, heme oxygenase-1; NQO1, NAD(P)H quinone oxidoreductase 1; AMPK, AMP-activated protein kinase; Nrf2, nuclear factor erythroid 2-related factor 2.

**Table 5:** Metabolic improvement

Assay	Effect and mechanism	Source and compounds	Author	Ref.
ICR mice with high-fat diet, wistar rats	Increase in fecal content in mice with high-fat diet and inhibition of pancreatic lipase activity in wistar rats	Chikusetsusaponins isolated from *P. japonicus* rhizomes	Han *et al.* (2005)	(64)
3T3-L1, diet-induced obesity-prone mice	Suppression of preadipocyte differentiation, adipogenetic transcription factors, PPAR-γ2, C/EBP and SREBP-1c, decrease in body weight gain and visceral fat tissue accumulation, amelioration of the plasma cholesterol concentration	Ethanol extract of *P. japonicus* flower buds	Watanabe *et al.* (2010)	(65)
Pancreatic lipase activity *in vivo*	Potential pharmacological effects on obesity and inhibitory effects against pancreatic lipase	Ethanol extract of *P. japonicus* (Siebold & Zucc.) Maxim. leaves and stems	Lee *et al.* (2012)	(66)
H4IIE, 3T3-L1,C2C12 cells, C57BL/6J mice	Modulation of glucose metabolism and activation of AMPK through mitochondrial respiration inhibtion	Petasin from *P. japonicus shoot*	Adachi *et al.* (2014)	(67)
3T3-L1 cell	Inhibition of adipogenesis, reduction in glucose uptake, inhibition of triglyceride accumulation, down-regulation of the expression of PPAR-γ and its target genes	*S*-Petasin isolated from *P. japonicus* rhizomes	Guo *et al.* (2019)	(68)

PPAR, peroxisome proliferator-activated receptor; C/EBP, CCAAT-enhancer-binding protein; SREBP-1c, sterol regulatory element-binding protein-1c; AMPK, AMP-activated protein kinase.

**Table 6:** Anti-cancer effect

Assay	Effect and mechanism	Source and compounds	Author	Ref.
P-388 lymphocyte leukemia cells	Tumor growth inhibition of picrasinoside B and tumor growth promotion of picrasin B	Picrasin B, picrasinoside A, and picrasinoside B isolated from *Picrasma quassioides* and fukinolide isolated from *P. japonicus*	Nadamitsu *et al.* (1985)	(71)
HUVEC	Anti-angiogenic effect via inhibition of DNA polymerase λ activity	Petasiphenol from *P. japonicus*	Matsubara *et al.* (2004)	(72)
Hep3B cells	Growth inhibition through inhibition of the Akt/mTOR and Wnt signaling pathways	95% Methanol extract of *P. japonicus* roots	Kim *et al.* (2015)	(73)
HeLa cells, xenografted mice	Growth inhibition, induction of apoptosis by upregulation of Bax and down-regulation of Bcl-2, activation of caspase-9, capspase-3, and PARP, reduction in tumor weight	70% Ethanol extract of the whole *P. japonicus* plant	Hwang *et al.* (2015)	(74)

HUVEC, human umbilical vein endothelial cells; PARP, poly (ADP-ribose) polymerase.

## Abbreviations

Aβ: amyloid β
ABTS^+^: 2-2'-azino-bis(3-ethylbenzothiazoline-6-sulfonic acid)
AD: Alzheimer’s disease
AMPK: AMP-activated protein kinase
BACE1: β-secretase 1
COX-2: cyclooxygenase-2
DPPH: 1,1-diphenyl-2-picrylhydrazyl
FRAP: ferric-reducing ability of plasma
HPLC: high-performance liquid chromatography
iNOS: inducible nitric oxide synthase
LC–MS/MS: liquid chromatography–tandem mass spectrometry
NO: nitric oxide
Nrf2: nuclear factor erythroid 2-related factor 2
ORAC: oxygen radical absorbance capacity
PCA: passive cutaneous anaphylaxis
PPAR: peroxisome proliferator-activated receptor
ROS: reactive oxygen species
TBARS: thiobarbituric acid-reactive substance
